# Traumatic Dental Injuries Among Young Orthodontic Patients in Public Dental Health Service: Patterns of Occurrence and Referral Routines

**DOI:** 10.1002/cre2.70404

**Published:** 2026-07-20

**Authors:** Dorina Sula Thelen, Ingrid Gramstad Skeie, Mahkameh Nicole Aria, Madeleine Misje Roman Beyene, Asgeir Bårdsen, Gerhard Sulo

**Affiliations:** ^1^ Oral Health Center of Expertise in Western Norway Bergen Norway; ^2^ Oral Health Center of Expertise Rogaland Stavanger Norway; ^3^ Department of Clinical Dentistry, Faculty of Medicine University of Bergen Bergen Norway; ^4^ Department of Global Public Health and Primary Care University of Bergen Bergen Norway

**Keywords:** adolescents, dental trauma, Norway, orthodontic treatment, referral routines, severity

## Abstract

**Objectives:**

Orthodontic treatment is performed between 6 and 20 years—a period in life carrying the highest risk for traumatic dental injuries. The presence of traumatic injuries leads to treatment modifications and may adversely impact orthodontic outcomes. Yet, the literature on the prevalence and characteristics of traumatic injuries among patients undergoing orthodontic treatment is sparse. This study aims to describe the burden and distributional patterns of traumatic injuries, and the quality of information provided by general dentists to specialists on them (i.e., referral practices) among children undergoing orthodontic treatment.

**Material and Methods:**

We included in the study 1529 participants (46.1% males, aged 7–18 years) undergoing orthodontic treatment in Western Norway, 2001–2018. Demographic, clinical, and other relevant information related to orthodontic treatment, as well as referral practices on traumatic injuries, were obtained from journals.

**Results:**

Overall, 16.5% of participants experienced traumatic injuries. The prevalence did not change over time or across age categories but was higher among males (OR = 1.81; 95% CI:1.38–2.39). Traumatic injuries were mostly mild (85%), involved often (79.8%) ≤ two teeth, typically maxillary central incisors, and occurred mostly at school (40%). Out of 201 participants with TDIs before commencement of orthodontic treatment, only 9% of them had adequate information on trauma in their electronic journals. This proportion was, however, higher (48.4%) when trauma was moderate or severe. The severity of trauma was strongly associated with the quality of referral routines (17.4 and 83.5 times more likely to have adequate referral, for moderate and severe injuries, respectively, compared to mild injuries).

**Conclusions:**

TDIs are common among patients receiving orthodontic treatment, with males being at higher risk. The severity of TDIs was strongly associated with referral routines, highlighting the need for improved referral practices to optimize information and subsequent treatment in this subgroup of patients.

## 1 Introduction

Traumatic dental injuries (TDIs) are a common health problem in children and adolescents (Andersson 2013; Glendor 2009; Petti et al. 2018). In Norway, the prevalence of TDI among 16‐year‐old adolescents was reported to be 16.4 (Bratteberg 2018), with milder injuries dominating the clinical presentation. The etiology of TDI is complex (Glendor 2009) and multifactorial (Magno 2020; Arraj et al. 2019).

Orthodontic treatment (OT) focuses on diagnosing, preventing, and correcting dental and facial irregularities to enhance oral function, esthetics, and overall health. Children and adolescents account for most orthodontic patients. In Norway, 21% of individuals aged 6–20 years receive OT (Ekornrud et al. 2019). The Public Dental Health Service (PDHS) in Norway provides treatment for TDI, offering free‐of‐charge dental services to children and adolescents until the age of 18 (Statistisk sentralbyrå 2020). Public general dentists or dental hygienists in Norway refer patients with malocclusion to an orthodontist (Helse‐ og omsorgsdepartementet 2021), often at early mixed dentition, and this timing coincides with the peak age of experiencing TDIs as well (Petti et al. 2018). Further, it has been shown that candidates for OT are at higher risk of experiencing TDIs (Borzabadi‐Farahani and Borzabadi‐Farahani 2011) and that increased overjet, inadequate lip coverage, and proclination of anterior teeth are common traits for patients with TDI and OT need (Koroluk et al. 2003; Kindelan 2008; Bauss et al. 2004; Borzabadi‐Farahani et al. 2010; Brin 2000; Schatz 2020).

The presence of TDIs challenges both the treatment plan and the outcomes of OT. Orthodontic movement of teeth affected by TDI is more prone to complications, such as adverse pulpal reactions and root resorption (Bakkari and Bin Salamah 2022; Bauss, Röhling et al. 2008; Bauss et al. 2009; Duarte 2023). The combination of TDI with orthodontic tipping renders teeth more susceptible to complications, especially to root resorption and loss of vitality (Duarte 2023; Brin 1991). Teeth with severe periodontal injury during OT and subsequent total pulp obliteration have an increased risk of pulp necrosis during additional OT stages (Bauss 2009). Orthodontic forces on a TDI tooth should be lightened or discontinued while the healing process occurs (Bakkari and Bin Salamah 2022). Therefore, obtaining clinical information on the diagnosis of TDI‐affected teeth through referral routines before initiating OT is crucial.

International studies focusing on OT of traumatized teeth revealed substantial variations in treatment procedures (Beyene 2023), recommended observation periods and management strategies (Rajab et al. 2024; Sandler 2019; Stučinskaitė 2023) as well as varying adherence to international guidelines and deviation from evidence‐based recommendations.

An issue raised is the quality of referral routines. A survey among Norwegian orthodontists reported that the quality of information referred from PDHS for TDI‐affected patients was perceived as inadequate (Beyene 2023). Another study from Belgium found that referral routines among general and pediatric dentists were only moderately influenced by the presence of TDI. Many dentists did not schedule additional follow‐up assessments during OT, largely due to limited access to clear clinical guidelines for managing these cases (Van Gorp 2019).

Information on the burden, characteristics, and distributional patterns of TDIs in orthodontic patients remains limited, constraining our understanding of the observed variations in OT approaches. To address this gap, the present study sought to: (i) quantify the burden and assess temporal trends in the distribution of TDIs; (ii) investigate patterns of occurrence and injury severity; and (iii) evaluate referral practices from general dentists and dental hygienists for children and adolescents referred for OT.

## 2 Methods

### 2.1 Study Population and Settings

The study was carried out in two regional odontological competence centers in Western Norway (Hordaland and Rogaland counties) during 2001–2018, which are part of the PDHS.

The PDHS offers free‐of‐charge dental treatment for all children and adolescents up to 18 years of age, except for OT, which is only partly financed by Helfo (The Norwegian Health Economics Administration). The PDHS has used electronic patient's dental records (EPDR) from Opus Dental since 1998 (Opus Systemer AS).

In total, 1529 patients, aged 7–18 years, who underwent OT and had complete information on their EPDR regarding OT and TDIs, were included in the study.

### 2.2 Data Content and Definitions

Information collected from orthodontic EPDRs for each study participant included (i) demographics (sex, age at OT commencement and county), (ii) orthodontic diagnosis and applied treatment (including duration in months), and (iii) presence of trauma (including type, localization, number of teeth involved, severity, place of occurrence, cause and time lag from its occurrence to the commencement of OT).

TDIs were classified based on the WHO classification system, modified by Andersson et al. (2019) and grouped in our study into “mild,” “moderate,” and “severe,” according to Skaare and Jacobsen (2003a). In addition to severity, patterns related to their cause, location, age at first trauma, and their seasonal distribution were analyzed.

Relevant information also included orthodontic diagnosis (Angle's classification), skeletal measurement (i.e., maxillo‐mandibular sagittal relation measured as ANB angel and classified as “neutral basal sagittal relation” [ANB = 0–4 degrees], “distal basal sagittal relation” [ANB > 4 degrees] and “mesial basal sagittal relation” [ANB < 0 degrees]), horizontal overjet (recorded in mm and categorized into “negative overjet”; < 0 mm, “normal overjet”; 0–6 mm and “exceed overjet”; ≥ 6 mm), type of OT (defined as “removable,” “fixed,” or “combination”), OT period (measured in months), and lip coverage (of the upper incisors before OT, classified as “adequate” if the lip covered the upper incisors in rest position or “inadequate” if the crown of incisors was exposed).

Lastly, in the referral form sent from general dentists and hygienists to orthodontists, we scrutinized the section providing information on prior TDIs (i.e., TDIs referral quality). TDIs referral quality was defined as “optimal” (when existing TDI diagnoses and time of occurrences were described in the referral form) and “not sufficient” [when only the presence of TDIs was mentioned briefly (without additional information on diagnosis and timing) or when the presence of TDIs was not mentioned at all).

### 2.3 Statistical Analysis

Continuous variables are presented as mean (SD) or median (interquartile range), and categorical variables as numbers and proportions. Independent sample *t*‐tests, Mann–Whitney or Kruskal–Wallis tests were used for comparisons of normally or skewed distributed continuous variables, while chi‐square test or Fisher's exact test were used to compare categorical variables. Logistic regression model was used to explore trauma occurrence as a function of study year, sex, and age of study participants. To explore the role of age, sex, study year, and severity of TDIs on the quality of referral routines, we fitted a Firth penalized logistic regression model. From each set of analyses, we reported odds ratios (OR) and corresponding 95% confidence intervals (Cis). Analyses were performed using STATA software, version 18.

### 2.4 Ethical Considerations

Data were collected through the EDRs from OHCE Western Norway, Hordaland, and OHCE Rogaland. The dataset was unidentifiable after it had been reviewed and stored in the safe databases handled by the PDHS in Hordaland and Rogaland. The Regional Committees for Medical and Health Research Ethics (REC) approved the study as a quality assurance project without further evaluation. The project was approved by the Norwegian Center for Research Data (NSD) for anonymous management of the collected data.

## 3 Results

### 3.1 Study Population

The characteristics of 1529 study participants (overall, and by presence of TDIs) are depicted in Table 1. Overall, the mean (SD) age was 12.6 (2.4) years, with males accounting for 46.1% of the study population. The OT lasted (median [IQR], 24 [17–32]) months. Of all patients, 10.9% were treated with removable orthodontic appliances, 75.2% with fixed orthodontic appliances, and 13.9% with a combination of both. Angle Class II classified occlusion was more frequent (58.4%), followed by Class I (29.5%) and Class III (12.1%) malocclusions. The majority had a normal horizontal overjet (62.4%), followed by an increased overjet (28.8%) and negative overjet (8.8%). Most patients (63.2%) had a “neutral” basal sagittal relation, followed by a “distal” basal sagittal relation (28.7%) and a “mesial” basal sagittal relation (7.6%). Lip coverage was absent in 17.4% of the study participants.

**Table 1:** Characteristics of orthodontic patients, 2001–2018; overall and by presence of traumatic dental injuries.

Characteristics of study participants	Total (*n* = 1529)	Without TDIs (*n* = 1277)	With TDIs (*n* = 252)	*p* value
Age, *mean (SD)*	12.6 (2.4)	12.6 (2.4)	12.4 (2.2)	0.301
Sex, *n (%)*				< 0.001
Males	705 (46.1)	558 (43.7)	147 (58.3)	
Females	824 (53.9)	719 (56.3)	105 (41.7)	
Treatment duration, *median (IQR)*	24 (17–32)	24 (17–32)	24 (17–32)	0.964
Type of treatment, *n (%)*				0.091
Removable/plate	166 (10.9)	145 (11.4)	21 (8.4)	
Fixed/fast	1147 (75.2)	962 (75.5)	185 (74.0)	
Combination	212 (13.9)	168 (13.2)	44 (17.6)	
Orthodontic diagnose				0.006
Angle class I	436 (29.5)	364 (29.4)	72 (30.0)	
Angle class II	863 (58.4)	710 (57.4)	153 (63.7)	
Angle class III	178 (12.1)	163 (13.2)	15 (6.3)	
* Missing*	*52*	*40*	*12*	
Overjet				< 0.001
Negative	129 (8.8)	118 (9.7)	11 (4.6)	
Normal	910 (62.4)	774 (63.4)	136 (56.9)	
Exceed	420 (28.8)	328 (26.9)	92 (38.5)	
* Missing*	*70*	*57*	*13*	
Basal sagittal relation				0.008
Neutral	910 (63.2)	777 (64.4)	133 (56.6)	
Mesial	117 (7.6)	102 (8.5)	15 (6.4)	
Distal	414 (28.7)	327 (27.1)	87 (37.0)	
* Missing*	*88*	*71*	*17*	
Lip coverage				0.041
Present	1170 (82.6)	996 (83.5)	174 (77.7)	
Absent	247 (17.4)	197 (16.5)	50 (22.3)	
* Missing*	*112*	*84*	*28*	

Abbreviations: IRQ, interquartile range; SD, standard deviation; TDI, traumatic dental injury.

Compared to participants without TDIs, those with TDIs were more often males (58.3% vs. 43.7%), had more often an angle Class II occlusion (63.7% vs. 57.4%), “increased” overjet (38.5% vs. 26.9%), and had more often absent lip coverage (22.3% vs. 16.5%) (Table 1).

### 3.2 Distribution of Traumas

In total, 252 study participants had experienced TDIs, either prior to (201) or during (51) OT, yielding a prevalence of 16.5%. Study participants in whom TDIs occurred before OT were older compared to those in whom TDI occurred during OT (mean [SD] age: 12.6 [2.1] vs. 11.7 [2.1]; *P*_comparison_ = 0.026) while no differences between groups were observed regarding sex (*P*_comparison_ = 0.534), trauma severity (*P*_comparison_ = 0.303), or study year (*P*_comparison_ = 0.431) (data not shown).

**Figure 1:**
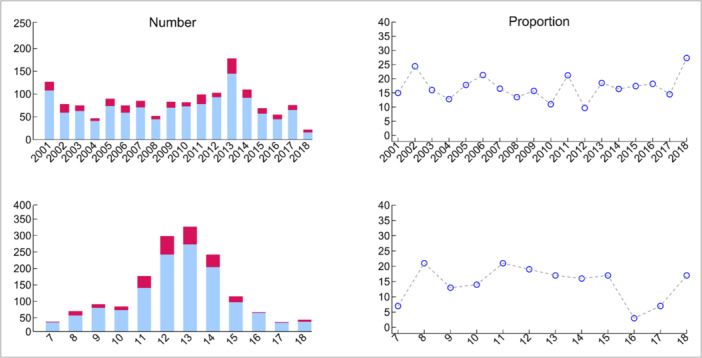
Number and proportion of study participants with traumatic dental injuries (TDI) by study year (upper panel) and age (lower panel).

The proportion of TDIs (within all patients receiving OT) varied widely (Figure 1, upper panel), without showing a clear pattern over time (*P*_linear trend_ = 0.76). Similarly, we did not observe a clear age pattern in the distribution of TDIs (*P*_linear trend_ = 0.42) (Figure 1, lower panel).

When all pre‐defined dimensions (study year, sex, and age of participants) were accounted for in the model, we observed no change in the odds of TDIs over time (OR = 1.01; 95% CI: 0.97–1.03) or with increasing age (OR = 0.96; 95% CI: 0.91–1.02). Males, on the other hand, had 81% higher odds (OR = 1.81; 95% CI: 1.38–2.39) of having experienced a TDI compared to females (Table 2).

**Table 2:** Association between dental trauma injuries and study year, sex, and age of study participants.

Characteristics	Odds ratio^a^ (95% CI)
Study year *(1 year increment)*	1.01 (0.97–1.03)
Sex	
Females	1 ^(reference)^
Males	1.81 (1.38–2.39)
Age *(1 year increment)*	0.96 (0.91–1.02)

Abbreviation: CI, confidence interval.

^a^ Obtained from logistic regression with all variables included in the model.

### 3.3 Characteristics of TDIs

**Table 3:** Characteristics of traumatic dental injuries: Overall and by sex.

Characteristics of traumas	Total (*n* = 252)	Men (*n* = 147)	Women (*n* = 105)	*p* value
Age, *mean (SD)*	12.4 (2.2)	12.6 (2.2)	12.1 (2.1)	0.076
Timing of trauma, *n (%)*				0.638
Before orthodontal treatment	201 (88.2)	117 (87.3)	84 (89.4)	
During orthodontic treatment	27 (11.8)	17 (12.7)	10 (10.6)	
Number of traumatized teeth, *n (%)*				0.446
1	105 (45.1)	57 (41.9)	48 (49.5)	
2	81 (34.7)	52 (38.2)	29 (29.9)	
3	20 (8.6)	10 (7.4)	10 (10.3)	
≥ 4	27 (11.6)	17 (12.5)	10 (10.3)	
Seasonality of TDIs, *n (%)*				0.177
Q_1_	48 (23.0)	22 (17.7)	26 (30.6)	
Q_2_	53 (25.4)	34 (27.4)	19 (22.4)	
Q_3_	49 (23.4)	32 (25.8)	17 (20.0)	
Q_4_	59 (28.2)	36 (29.0)	23 (27.0)	
* Missing*	*43*	*23*	*20*	
Place of TDIs’ occurrence, *n (%)*				0.04
Home	35 (19.4)	18 (17.0)	17 (23.0)	
Street	34 (18.9)	20 (18.9)	14 (18.9)	
School	72 (40.0)	43 (40.6)	29 (39.2)	
Sport	21 (11.7)	18 (17.0)	3 (4.1)	
Other	18 (10.0)	7 (6.6)	11 (14.8)	
* Missing*	*72*	*41*	*31*	
Cause of trauma, *n (%)*				0.135
Fall	93 (51.4)	48 (45.2)	45 (60.0)	
Collision	57 (31.5)	36 (34.0)	21 (28.0)	
Violence	10 (5.5)	8 (7.6)	2 (2.7)	
Other	21 (11.6)	14 (13.2)	7 (9.3)	
* Missing*	*71*	*41*	*30*	

*Note:* Q1 to Q4: Quarters one to four.

Abbreviations: SD, standard deviation; TDI, traumatic dental injury.

The mean (SD) age of participants with an injury was 12.4 (2.2) years. Most TDIs (88.2%) occurred before the start, while 11.8% took place during OT (Table 3). In most cases, traumas involved either a single tooth (45.1%) or two teeth (34.7%). The seasonal distribution of TDIs was balanced. School was the most common location for TDIs, accounting for 40.0% of cases. Falls were the predominant cause of TDIs, accounting for 51.4% of cases, followed by collisions (31.5%).

Males were slightly older than females at the age of TDI. Further, sex differences were observed regarding place of TDI's occurrence; sport‐related injuries were more prevalent in males than females (17.0% vs. 4.1%), whereas females had more often than males suffered a TDI at home (23.0% vs 17.0%) and other places (14.8% vs. 6.6%) (*p* = 0.04).

### 3.4 Severity of Traumas

**Table 4:** Severity of traumatic dental injuries at the tooth level.

Type of trauma	Maxillary central incisors	Maxillary lateral incisors	Maxillary canines	Mandibular central incisors	Mandibular lateral incisors	Mandibular canines	Total
Mild traumas^a^	211	54	3	47	16	5	336
Moderate traumas^b^	20	9	—	1	—	—	30
Severe traumas^c^	12	6	1	5	6	—	30
Total	243	69	4	53	22	5	396

^a^ Enamel or enamel‐dentin fracture, concussion, subluxation, enamel‐dentin fracture with concussion, enamel fracture with subluxation, enamel‐dentin fracture with subluxation, and enamel fracture with concussion.

^b^ Complicated crown fracture, uncomplicated crown‐root fracture, and horizontal root fracture.

^c^ Complicated crown‐root fracture, root fracture with luxation, extrusion, lateral luxation, intrusion, avulsion, subluxation with lateral luxation, enamel‐dentin fracture with extrusion, and root fracture with extrusion.

The distribution of severity of TDI on the affected teeth (*n* = 396) was as follows: 336 mild (85%), 30 moderate (7.5%), and 30 severe (7.5%) (Table 4). If a tooth had several TDI diagnosis, only the most severe diagnosis was recorded. The most affected teeth were maxillary central incisors, followed by maxillary lateral incisors.

### 3.5 Adequacy of Referral Routines

A total of 201 participants had experienced TDIs prior to the initiation of OT (Tables 5 and 6). Of these, only 18 (9.0%) had adequate TDI‐related information documented in their EPDRs. The proportion was higher among male compared to female study participants (10.4% vs. 7.1%). Among the 31 participants with moderate or severe trauma, adequate information was provided by general dentists or hygienists and recorded in the EPDR in 48.4% of cases. This proportion was again higher in males compared to females (52.4% vs. 40.0%) (Table 5).

**Table 5:** The TDIs’ referral quality: Overall and by TDIs severity.^a^

Quality of referral routines	All traumas (*n* = 201)			Moderate to severe traumas (*n* = 31)		
	Total (*n* = 201)	Male (*n* = 117)	Female (*n* = 84)	Total (*n* = 31)	Male (*n* = 21)	Female (*n* = 10)
Not sufficient	172 (85.6)	100 (85.4)	72 (85.8)	12 (38.7)	8 (38.1)	4 (40.0)
Good	18 (9.0)	12 (10.4)	6 (7.1)	15 (48.4)	11 (52.4)	4 (40.0)
Missing	11 (5.4)	5 (5.2)	6 (7.1)	4 (12.9)	2 (9.5)	2 (20.0)

^a^ Only traumas occurring prior to the commencement of orthodontic treatment.

In the multivariable model, the severity of TDI was strongly associated with referral routines, though the estimates were quite imprecise due to small sample size (Table 6). Compared to mild TDIs, moderate and severe traumas had respectively 17.38 (OR = 17.38; 95% CI: 3.11–97.38) and 83.52 (OR = 83.52; 95% CI: 14.86–469.14) times higher odds of being adequately referred in the age, sex, and study year‐adjusted model. Further, we observed a slight, but significant improvement (OR = 1.06; 95% CI: 1.02–1.07) in the adequacy of referral over the study period. No statistically significant associations with adequacy of referral routines were observed for the time lag between trauma occurrence and OT.

**Table 6:** Association between adequacy of referral routines and participants age, sex, trauma severity, and its timing.

Characteristics	Odds ratio^a^ (95% Cl)
Age, *(1 year increment)*	1.18 (0.91–1.54)
Study year	1.04 (1.02–1.07)
Sex	
Female	1 ^(^^reference)^
Male	0.98 (0.27–3.52)
Trauma severity	
Mild	1 ^(^^reference)^
Moderate	17.38 (3.11–97.38)
Severe	83.52 (14.86–469.45)
Time lag *(1 month increment)* between TDI and orthodontic treatment	1.11 (0.78–1.59)

Abbreviation: CI, confidence interval.

^a^ Obtained from the Firth penalized logistic regression with all variables included in the model.

## 4 Discussion

TDIs were observed in 16.5% of participants, with the majority occurring prior to the initiation of OT. TDIs were more common among males and were associated with Angle Class II malocclusion, increased overjet, and absent lip coverage. No temporal trend in TDI occurrence was identified over the study period, nor was there a significant association with age. Most injuries were mild in severity, typically affecting one or two teeth—predominantly maxillary incisors—and were most frequently caused by falls, often occurring in school settings. Documentation and referral practices were limited overall, with adequate TDI‐related information recorded in a small proportion of cases, although higher severity injuries were substantially more likely to be adequately referred. A modest improvement in referral adequacy was observed over time.

### 4.1 Prevalence of TDIs Among Children and Adolescents Receiving Orthodontic Treatment

The prevalence and characteristics of TDIs reported in the general population (Kaczmarek et al. 2019; Locker 2005; Soriano 2007) may not directly correspond to those observed among children and adolescents of similar age receiving OT. The extent to which these differences reflect characteristics specific to orthodontic patients remains uncertain.

Very few studies have focused on burden and TDIs’ characteristics among orthodontic patients. A study including 1367 consecutive orthodontic patients from a private practice in Germany (1998–2002), reported a TDI prevalence of 10.3% (Bauss et al. 2004), while the prevalence was higher (14.3%) among 121 consecutive patients visiting a university orthodontic clinic center in Italy (2012–2019) (Di Venere et al. 2020). The differences in the TDI prevalence reported from these two international studies and our estimates can be attributed to variations in the age group studied, the TDI definition, and data collection modalities. In the Bauss et al. (2004) study, participants age spanned from 6 years to 55 years, while Di Venere et al. (2020) allowed in their study for the inclusion of TDIs in deciduous teeth as well. Moreover, both studies relied on questionnaires, which are subject to recall bias and could underestimate the true prevalence of TDI. Conversely, our study utilized longitudinal dental record data, potentially offering more accurate and comprehensive information on TDI occurrence.

### 4.2 Time Trends and Sex Differences

Our study did not show any clear increase or decline in TDI prevalence over time, while TDI was more prevalent among boys compared to girls, even after adjusting for age and study year. The two directly comparable studies (Bauss et al. 2004; Di Venere et al. 2020) did not analyze time trends in TDI prevalence. However, they reported double as high crude TDI prevalence among males compared to females: (18.1% vs. 9.7%) (Di Venere et al. 2020) and 14.0% vs. 7.1% (Bauss et al. 2004). Earlier publications on the general population have also reported a male‐to‐female ratio ranging from 1.3 to 1 and up to 2.5 to 1 (Bratteberg 2018; Lam 2016; Mendez 2025). Reports from Norway confirmed international findings of a higher prevalence of TDI among boys compared to girls (Bratteberg 2018). It has been suggested that males have a higher tendency toward contact sports and aggressive behavior while engaging in sports and recreational activities.

### 4.3 TDIs Characteristics and Influencing Factors

A significant difference was found between Class I, CL II, and Class III Angle's classification (malocclusion) as well as with a distal skeletal Class II sagittal relationship (ANB angle > 4°), in relation to the occurrence of TDIs, indicating that a greater percentage of TDIs occurred in individuals with Class II relationships and with a distal sagittal relation Class II. Di Venere et al. (2020) found a significant correlation between skeletal Class II and the increased risk of traumatic injury in an orthodontic population. These findings were in accordance with other studies involving a more general population (Borzabadi‐Farahani et al. 2010; Kania 1996).

Our study findings point to an association between increased overjet and the likelihood of experiencing TDI. Studies on an orthodontic patient's population are in accordance with these findings (Di Venere et al. 2020). However, Bauss, Freitag et al. (2008) found that increased overjet > 3.0 mm and inadequate lip coverage increase the risk and severity of incisor trauma. They concluded that early OT might prevent dental trauma in these patients. In our study, an overjet of ≥ 6 mm was classified as increased, as this threshold qualifies a child for treatment reimbursement from the Norwegian government due to the risk of TDI. This finding is also consistent with prior research (Kania 1996; Bauss, Freitag et al. 2008), which indicates that this dental trait may increase susceptibility to such injuries.

Moreover, a meta‐analysis by Arraj et al. (2019) identified a slightly lower threshold for TDI risk, pointing to an overjet of 5 mm as a significant marker. This suggests that even slight elevations in overjet measurements can be important for assessing the risk of TDI.

The suggestion for OT to manage increased overjet depends on several considerations, such as the patient's activity level, age, and gender (Brin 2000; Burden 1995). This personalized approach highlights the necessity of customized treatment plans within orthodontics.

Previous evidence (Koroluk et al. 2003) suggests that early orthodontic intervention—particularly in children with increased overjet, may reduce the risk of new TDIs. A Cochrane review (Thiruvenkatachari 2015) indicated that two‐phase treatment may reduce new incisal TDIs compared with treatment initiated in adolescence. However, our study lacks follow‐up data on treatment timing, treatment phases, and subsequent TDIs, and therefore cannot evaluate this question directly.

The maxillary central incisors were identified as the most affected teeth, which aligns with findings in the existing literature (Bratteberg 2018; Lam 2016; Glendor 2008). The flexible attachment of the lower jaw to the skull allows it to absorb impacts, reducing the force transmitted to the mandible. Additionally, because Class III malocclusions are relatively rare in the present study and naturally protect the lower front teeth (mandibular incisors), injuries to the upper front teeth (maxillary incisors) occur more frequently than injuries to the lower front teeth (Borzabadi‐Farahani and Borzabadi‐Farahani 2011).

Our study did not find (statistically) significant differences in the use of fixed/fast appliances between patients with and without TDIs. While the presence of TDI did not systematically alter the broad choice of appliance type in our study, its influence is expressed at the level of biomechanics. For teeth with TDIs, clinicians may need to reduce overall force magnitude, use more flexible archwires, avoid or postpone intrusive or torque‑intensive movements, segment arches to exclude compromised teeth, or modify anchorage strategies (Day 2020). Additionally, the timing and sequencing of mechanics may be adjusted to allow for pulpal and periodontal healing, with closer radiographic and clinical monitoring and possible coordination with endodontic or restorative procedures. None of these tooth‑ or segment‑specific biomechanical modifications is captured by simply looking at the treatment uptake type. Future, prospective studies with a focus on biomechanical strategies, timing of force application, and long‑term pulpal and periodontal outcomes are needed to clarify how TDIs influence orthodontic management in everyday practice.

### 4.4 Distribution of Traumas and Their Severity

In our study, 45.1% of all TDIs involved a single tooth, while an additional 54.7% involved ≥ 1 teeth. This differs from Bauss et al. (2004), who reported that TDIs involved only one tooth in 53.9% of cases. Two earlier reviews reported that most TDIs involved only one tooth (Lam 2016; Glendor 2008). Monitoring these patterns over time is important, as they may be influenced by opposing factors, including increased participation in activities such as sports on the one hand, and improved awareness of risk and use of protective measures on the other.

Like previous publications (Bauss et al. 2004; Di Venere et al. 2020), the absolute majority of TDIs teeth in our study were maxillary central incisors. Orthodontic patients, who typically exhibit a higher prevalence of prominent upper teeth, a characteristic of Class II Division 1 malocclusion, which is one of the most common in Scandinavia (Josefsson et al. 2007), support the result of the present study. The increased occurrence of prominent teeth may also contribute to the higher frequency of multiple teeth involved in TDIs.

Like in the Bauss et al. (2004) study, milder TDIs generally dominated the picture in our study as well. However, we observed a higher prevalence of severe injuries (7.5%), compared to previous studies in Norway among the general population of children and adolescents (Bratteberg 2018; Skaare and Jacobsen 2003b). Patients with prominent upper incisors and inadequate lip coverage may tend to experience more severe dental traumatic injuries because of the increased exposure and vulnerability of the teeth and supporting structures (Brin 2000). The prominent incisors are more protruded, making them more susceptible to direct trauma during accidents or impacts. Additionally, inadequate lip coverage fails to provide a protective barrier, reducing natural cushioning and increasing the likelihood of injury from external forces. This combination of protrusion and insufficient lip protection may result in a higher risk of severe injuries such as fractures, avulsions, or soft tissue damage.

### 4.5 Referral Routines

To our knowledge, orthodontic referral routines for patients with a history of TDI have not previously been objectively investigated in Scandinavia. However, a survey among Norwegian orthodontists found that referral information from the PDHS for TDI‐affected patients was often perceived as inadequate (Beyene 2023)—though the objectively measured indicators of referral quality were not dealt with. A study from Belgium found that referral decisions by general and pediatric dentists were only moderately influenced by the presence of TDI, and many did not schedule additional follow‐up assessments during OT, nor were they aware of specific clinical guidelines for managing such cases (Van Gorp 2019).

A key issue highlighted by our study is the limited availability of information regarding the diagnosis, timing, treatment, and prognosis of TDIs. Referral information was more complete among patients with severe TDIs, possibly because such injuries are more consistently recorded or more readily recognized by dentists and dental hygienists. However, improving referral quality must also include less severe TDIs, as these may still affect OT planning, follow‐up, and outcomes. In selected cases, leaving a traumatized tooth off the archwire may be advantageous, particularly after recent TDIs, as it can reduce pressure on the injured tooth and surrounding tissues and allow healing and stabilization before orthodontic forces are applied (Kindelan 2008; Andreasen et al. 2019). Orthodontic movement of previously traumatized teeth may also increase the risk of complications, including pulp obliteration, pulp necrosis, and root resorption (Bauss, Röhling et al. 2008; Bauss et al. 2009; Malmgren 1982). Furthermore, depending on TDI severity, a period of observation is required after trauma to allow healing of the periodontal ligament and pulp before OT is initiated (Kindelan 2008; Day 2020).

### 4.6 Study Strengths and Limitations

Strengths of this study include the use of the WHO classification system, adapted by Andersson et al. (2019) enabling consistent diagnoses and comparability with other studies. Standardized data collection through an established organizational structure, EPDRs, and a dedicated TDI form ensured reliable, valid clinical information with minimal recall bias, thereby enhancing the validity and robustness of the findings.

When interpreting our results, it should be considered that the study population represents only a subset of orthodontic patients, specifically those treated within the PDHS. Consequently, we cannot confidently generalize our findings to all orthodontic patients in Norway, including those treated by private orthodontists. Lastly, the study lacks more recent data, which would have been valuable for assessing whether the trends observed in the current analyses have persisted over time.

## 5 Conclusions

TDIs affected 16.5% of children and adolescents undergoing OT. Traumas occurred predominantly before OT initiation, were mainly mild in nature, involved mostly maxillary incisors, and were more frequent among males. Overall, documentation and referral of TDIs were inadequate, although the adequacy increased with injury severity and showed slight improvement over time. These findings provide baseline information on traumas in this subset of children and adolescents, namely, those undergoing OT. They underscore the need for more consistent documentation and appropriate referral pathways, while also highlighting the need for further, preferably prospective, studies examining the significance of TDIs for OT routines and treatment outcomes.

## Author Contributions

Dorina Sula Thelen initiated the study, drafted the manuscript, and participated in data analyses. Ingrid Gramstad Skeie and Mahkameh Nicole Aria contributed substantially to data collection, study facilitation, preparation of the data/material, and technical support. Madeleine Misje Roman Beyene and Asgeir Bårdsen contributed to manuscript drafting, interpretation of the findings, and contextualization of the results within the existing literature. Gerhard Sulo contributed to study design, supervised the work, contributed to data analyses and presentation, as well as interpretation of the findings. All authors critically reviewed and approved the final manuscript before submitting it.

## Funding

The authors have nothing to report.

## Ethics Statement

Data were collected through the EPDR, OHCE Western Norway, Hordaland, and OHCE Rogaland. The Regional Committees for Medical and Health Research Ethics (REC) approved the study as a quality assurance project without further evaluation. The project was approved by the Norwegian Center for Research Data (NSD) for anonymous management of the collected data.

## Consent

The authors have nothing to report.

## Conflicts of Interest

The authors declare no conflicts of interest.

## Data Availability

The data underlying this study are not publicly available due to privacy, ethical, and legal restrictions. Access may be granted after application to the relevant registry/data owners and approval from the appropriate authorities. The dataset was unidentifiable after it had been reviewed and stored in the safe databases handled by the Public Dental Health Service in Hordaland and Rogaland.
